# A case report of Gorham-Stout disease diagnosed during the course of recurrent meningitis and cholesteatoma

**DOI:** 10.1186/s40463-020-00412-x

**Published:** 2020-04-16

**Authors:** Makoto Hosoya, Naoki Oishi, Jun Nishiyama, Kaoru Ogawa

**Affiliations:** Department of Otorhinolaryngology-Head and Neck Surgery, Keio University School of Medicine, 35 Shinanomachi, Shinjuku-ku, Tokyo, 160-8582 Japan

**Keywords:** Gorham-Stout disease, Cholesteatoma, Temporal bone, Osteolysis, Cerebrospinal fluid leakage

## Abstract

**Background:**

Gorham-Stout disease is a rare bone disorder. Here, we present a case of Gorham-Stout disease diagnosed during follow-up of a patient with cholesteatoma; the disease affected the temporal bone and other sites of the skull. To the best of our knowledge, this is the first report of Gorham-Stout disease diagnosed with recurrent cerebrospinal leakage after surgery to treat cholesteatoma.

**Case presentation:**

A 25-year-old male patient re-presented to our department for the first time in 7 years with otorrhea in the right ear and recurrent meningitis. The patient had a history of multiple surgeries for cholesteatoma and suffered from recurrent cerebrospinal fluid leakage, which initially was thought to be caused by recurrence of cholesteatoma. Therefore, skull base reconstruction was planned. However, the underlying cause was identified eventually as defects in the temporal bone caused by massive osteolysis due to Gorham-Stout disease. Skull base reconstruction was abandoned because the osteolysis was considered to be progressive. Conservative treatment with infectious control was implemented as an alternative.

**Conclusion:**

This case describes unusual temporal bone osteolysis after cholesteatoma surgery and the importance of considering the possibility of multiple concurrent diseases in such individuals. The distinguishing features of this case are the fact that the temporal bone had disappeared, and deconstruction was complicated by infection and inflammation caused by cholesteatoma, surgical invasion, and Gorham-Stout disease. Appropriate diagnosis saved the patient from ineffective multiple surgeries for cerebrospinal fluid leakage or cholesteatoma, and improved his quality of life.

## Background

Gorham-Stout disease is a rare bone disorder, the etiology of which is not fully understood [1]. This disease is accompanied by severe symptoms and has a variable prognosis. Pulmonary involvement along with chylothorax or spinal involvement may confer a poor prognosis, sometimes leading to death [2, 3]. In other cases, lesions may remain stable for long periods of time. The average mortality rate is 13% [4]. The etiology and clinical presentation of patients are poorly defined and, therefore, management remains a challenge. This disease typically affects the long bones and occasionally the temporal bones, although these cases are extremely rare. To date, 23 cases of Gorham-Stout disease affecting the skull base have been reported, of which 22 showed involvement of the temporal bone [5].

Here, we present a case of Gorham-Stout disease significantly affecting the temporal bone and other sites of the skull, in a patient with a history of cholesteatoma and presenting with recurrent cerebrospinal fluid (CSF) leakage and meningitis. To the best of our knowledge, this is the first report of Gorham-Stout disease diagnosed in a patient with recurrent CSF leak after surgery to treat cholesteatoma.

## Case presentation

A 25-year-old male patient re-presented to our department for the first time in 7 years with otorrhea in the right ear and recurrent meningitis. He had been treated for meningitis in another hospital and had experienced fever and seizures two weeks before visiting our hospital. The patient had a history of multiple surgeries for cholesteatoma and had been treated for severe otitis media at the age of 2 years, at which point right temporal bone osteolysis was identified. He subsequently suffered from recurrent meningitis and underwent several brain abscess drainage procedures. At ten years of age right temporal bone surgery was performed to rebuild the separation between the middle ear and lateral temporal lobe. At 16 years, he was diagnosed with cholesteatoma, and radical mastoidectomy was performed. Further details of these operations, performed at other hospitals, were not available. We were unable to obtain detailed past medical records regarding treatment (including surgical procedures) of his cholesteatoma, and we could not estimate whether the past intervention of the cholesteatoma was appropriate. When the patient was 17 years old, he visited our department for the first time to undergo assessment for controlling cholesteatoma, recurrent meningitis, and otalgia. First, tympanoplasty was performed to assess the disease and remove residual cholesteatoma from the middle ear; cholesteatoma was diagnosed by histopathological analysis. It was not possible to determine whether the cholesteatoma was a residual congenital cholesteatoma or a secondary cholesteatoma formed as a result of osteolysis of the external auditory canal or middle ear. At that time, we hesitated a more extensive procedure because of his age and limited information about his past surgical interventions. Meningitis and otalgia were subsequently controlled. However, after six months, when he was 18 years old, the patient experienced another episode of meningitis. At that time, he was also suffering from an epidural abscess, which was drained during hospitalization. He attended our department until he was 19 years old, at which point he chose to discontinue the hospital visits.

**Fig. 1:**
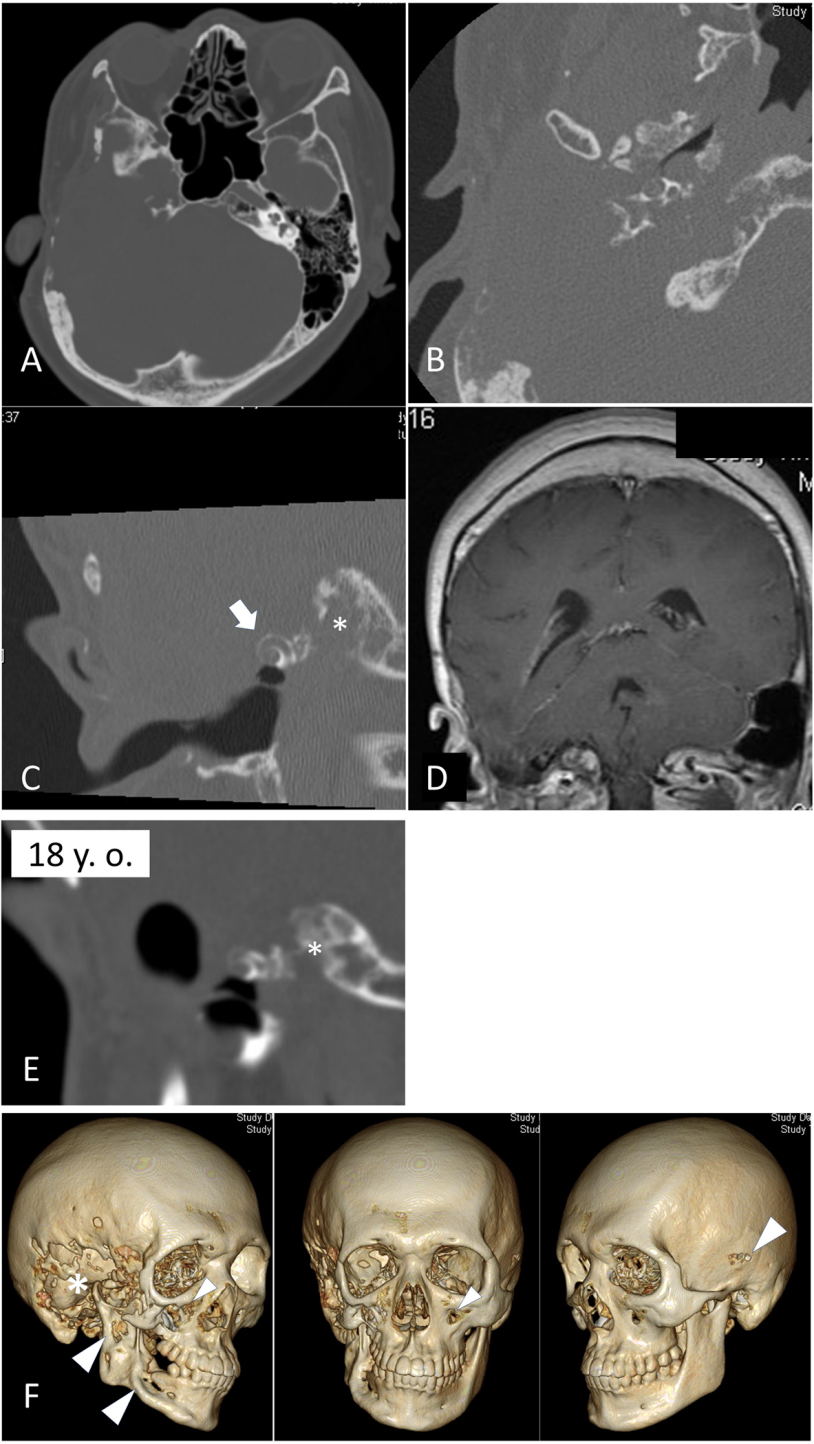
CT scans and MRI showing massive osteolysis of the skull bones. **a**−**c** The CT scans show that despite osteolysis affecting most of the temporal bone, the cochlear bony capsule was preserved and is almost intact (arrow in **c**). MRI revealed that the temporal lobe of the brain had herniated into the melted mastoid (**d**). The CT scans reveals progressive osteolysis of the temporal bone (asterisk in c and **e**) comparing with previous imaging obtained when he was 18 years old (**e**). 3D reconstructed CT clearly shows osteolysis of the skull bones; in addition to the right temporal bone (*), other affected bones include the mandibular bone, zygomatic bone, and contralateral temporal bone (arrowheads in **f**)

When the patient presented at our department again at the age of 25 years, computed tomography (CT) imaging revealed massive osteolysis of the temporal bone (Fig. 1 a−c). Notably, while most of the temporal bone had disappeared, the cochlear bony lateral wall was relatively well preserved, and his hearing by bone conduction had not been completely lost. Magnetic resonance imaging (MRI) revealed that the temporal lobe of the brain had herniated into the mastoid (Fig. 1d). Compared with a previous CT scan image (Fig. 1e), there is clear progression of boney destruction during a time when the patient did not undergo any surgery to the temporal bone. At this point, we chose an observational approach as otorrhea had spontaneously resolved. However, during the patient’s follow-up, otorrhea recurred, and cerebrospinal otorrhea was suspected. The patient was hospitalized and treated conservatively with antibiotics. Once again, the otorrhea resolved spontaneously with 2 weeks of bed rest, and skull base reconstruction was planned to prevent recurrence.

**Fig. 2:**
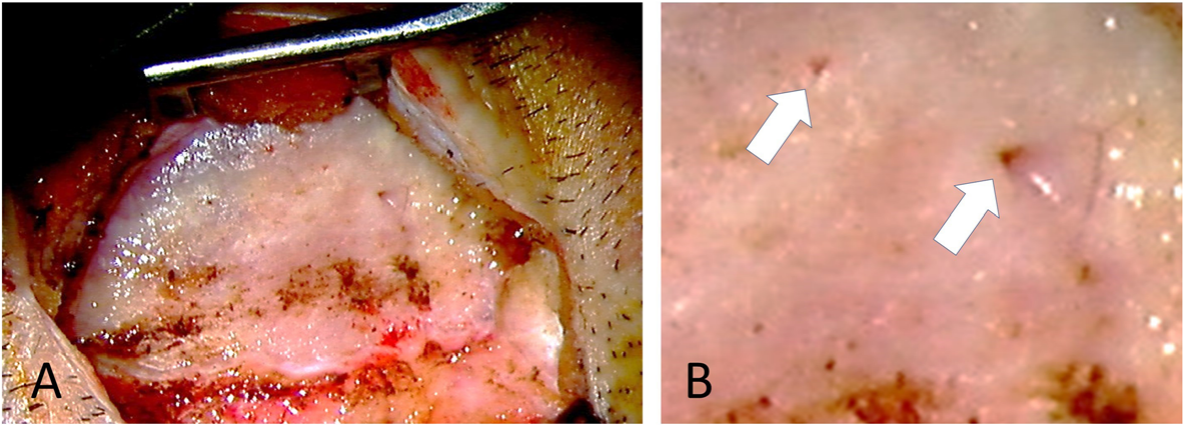
Intraoperative findings. Cerebrospinal fluid leakage from the abnormal pores is evident (**b**, arrows). **b** is a magnified image

**Fig. 3:**
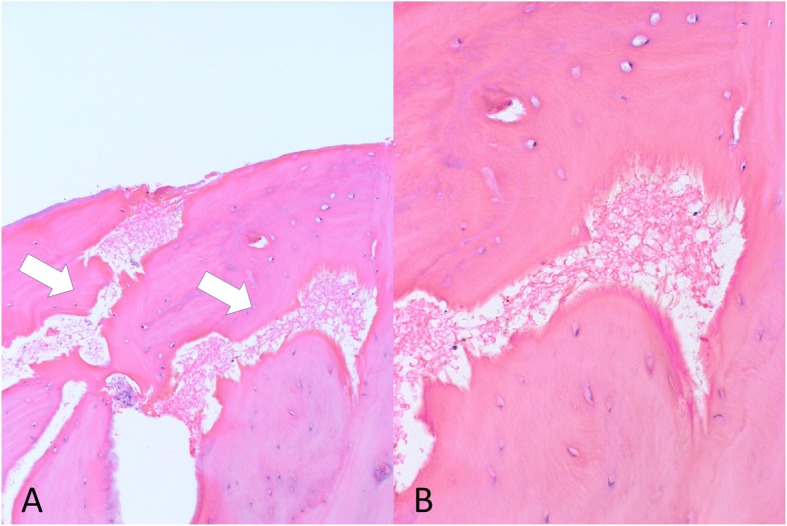
Histopathological findings. Formation of abnormal bone slits in bones that have maintained normal bone remodeling. Bone structures have been resorbed and replaced by thin- walled endothelium- lined capillaries of vascular or lymphatic origin (**a**, arrows). **b** A magnified image

?A3B2 twb=.27w?>Pre-operative CT imaging with three-dimensional reconstruction clearly revealed not only temporal bone osteolysis but also spotted osteolysis of other bones of the skull, including the mandibular bone, zygomatic bone, and contralateral temporal bone (Fig. 1f). At this point, Gorham-Stout disease was suspected for the first time. To support the diagnosis of Gorham-Stout disease (by ruling out other possible differential diagnoses), we performed temporal bone biopsy under general anesthesia. An incision was made on the post-auricular scar from previous surgery. When the periosteum flap was created, an unexpected massive serous leakage was encountered, spouting from abnormal pores or lytic lesions in the temporal bone (Fig. 2), which were similar to those described in previous reports [6]. Part of the temporal bone appeared blue in color, suggesting resorption. Samples were quickly collected from the bone surrounding the pores, and serous leakage (which appeared to be CSF) was stopped using fibrin glue and absorbable hemostat. There are no signs of recurrence of cholesteatoma at that time. Histopathological analysis revealed abnormal bony structures and accompanying small slits lined by epithelial cells (Fig. 3). The findings ruled out other diagnoses, and were supportive of a diagnosis of Gorham Stout Disease in conjunction with the clinical and radiographic information. We concluded that the progressive osteolysis was far more likely to have been caused by Gorham-Stout disease rather than cholesteatoma. Skull base reconstruction was abandoned as osteolysis was considered to be progressive. Conservative treatment with infectious control was implemented as an alternative.

During the first year of follow-up, he suffered a mild headache on one occasion, leading to an unscheduled visit. He was treated with intravenous antibiotics at an outpatient clinic to prevent the development of severe meningitis. At this point, no apparent CSF leakage was observed, no hospitalization was required and his headache had resolved within a few days. After that, there was no need to visit the hospital, except for follow-up once every few months. He was followed every two or 3 months for the next 1 year, during which time there were no signs of CSF or cholesteatoma recurrence.

We think that he has little risk of recurrence of the cholesteatoma because there has been no clear evidence of recurrence of cholesteatoma since the age of 18. However, this is an extremely rare case. CT imaging with three-dimensional reconstruction has been taken in every six months to assess the progression of osteolysis of the temporal or other bones of the skull and to watch out recurrent cholesteatoma. He remained well at the time of his last follow up. His quality of life has been maintained.

## Discussion

Gorham-Stout disease is a rare condition that is difficult to diagnose, as there is no specific test or procedure. Diagnosis is made by excluding other diseases, particularly lymphadenopathies, bone manifestations of systemic diseases, or other neoplastic lesions [7]. Previous studies proposed the following criteria to establish a diagnosis of for disease: 1) positive biopsy for presence of angiomatous tissue; 2) absence of cellular atypia; 3) minimal or no osteoblastic response and absence of dystrophic calcification; 4) evidence of local, progressive osseous resorption; 5) no expansile, nonulcerative lesion; 6) absence of visceral involvement; 7) an osteolytic radiographic pattern; and 8) no evidence of hereditary, metabolic, neoplastic, immunologic, or infectious etiology [8]. Despite variations in the histological appearance of this disease, typical histological findings reveal resorbed bone structures, which are replaced by thin-walled endothelium-lined capillaries of vascular or lymphatic origin [7]. A biopsy is also helpful for ruling out other entities during differential diagnosis.

To the best of our knowledge, this is the first case of Gorham-Stout disease accompanied by cholesteatoma; in several other cases, cholesteatoma was raised as a differential diagnosis alongside osteoradionecrosis, benign tumors (e.g., paragangliomas), and malignant and metastatic disease. In this paper, we made a diagnosis of Gorham-Stout disease based on radiological findings after excluding other differential diagnoses. The diagnosis was supported by histological findings. In this case, a characteristic osteolytic lesion was evident in the bones of the skull, including on the contralateral side that had not been affected by previous surgical procedures for cholesteatoma. Differential diagnoses, including neoplastic tumors or infectious diseases, were excluded after histological inspection. We noted the formation of abnormal bone slits in bones that had maintained normal bone remodeling. This histological finding supported our diagnosis as Gorham-Stout disease.

Simon et al. have published the largest case series of Gorham-Stout disease to date and reported a mean age at diagnosis of 3.5 years (range: 0–10) [5]. While most cases are diagnosed before adolescence, one case of Gorham-Stout disease diagnosed in a patient over the age of 50 years has been reported [9]. In the current case, the patient was 26 years old at diagnosis. He had undergone multiple surgeries for cholesteatoma, and therefore temporal bone osteolysis was initially thought to be the result of recurrent cholesteatoma; this surgical history delayed the diagnosis of Gorham-Stout disease. Regarding recurrent CSF leakage, Simon et al. reported that middle ear CSF leakage was observed in 13 of the 23 cases of skull base Gorham-Stout disease (57%) and many suffered from recurrent meningitis. In the current case, the patient also suffered from CSF leakage and recurrent meningitis. This recurrent CSF leakage was initially thought to be caused by the recurrence of cholesteatoma. However, eventually, the underlying cause was identified as defects in the temporal bone caused by massive osteolysis as a result of Gorham-Stout disease. Our case suggests that other osteolytic diseases, including Gorham-Stout disease, should be suspected early in the diagnostic process when encountering patients with cholesteatoma with unexplained massive osteolysis or recurrent CSF leakage.

Treatment of Gorham-Stout disease includes surgical intervention, medical treatment, and radiation therapy [3, 7]. Surgical intervention is mainly performed to prevent CSF leakage caused by skull base osteolysis. Medical treatment includes the use of bisphosphonates, interferon, bevacizumab, propranolol, steroids, vitamin D, calcitonin, and sirolimus. Although these treatments can improve the condition of some patients, there is no widely accepted standard of treatment for Gorham-Stout disease. In our case, the patient had experienced recurrent CSF leakage, but there was no obvious leakage point, and the condition could be managed with infection control and bed rest. Aggressive skull base reconstruction would have been challenging, and the benefits of this approach would not have outweighed the potential risk. Recurrence of CSF leakage would occur despite aggressive surgical repair unless the underlying osteolytic lesion was stabilized. Furthermore, even if the skull base was successfully reconstructed, we were afraid that meningitis would recur due to the abnormal pores found in the temporal bone, as observed during surgery (Fig. 2b), which will allow minor subcutaneous infections to be transmitted directly through the temporal bone. In addition, an observational approach was chosen because the patient did not want to commit to frequent hospital visits.

When faced with spontaneous CSF leakage or progressive osteolysis after cholesteatoma surgery, a surgical examination of the temporal bone is usually planned, regardless of the apparent recurrence of cholesteatoma. However, in this case, we found that the symptoms were caused by Gorham-Stout disease, not cholesteatoma. Thus, we concluded that recurrent surgical inspection of the temporal bone was inappropriate in this case.

## Conclusions

Here, we present an extremely rare case of Gorham-Stout disease, diagnosed with recurrent cerebrospinal leakage after surgery for cholesteatoma. Osteolysis of the temporal bone is associated with several diseases, including cholesteatoma, osteoradionecrosis, benign tumors (such as paragangliomas), and malignant and metastatic disease. Osteolysis of skull bones can also be caused by Gorham-Stout disease. Our case shows that temporal bone deconstruction can occur in the presence of multiple concurrent diseases. The distinguishing feature of this case is the fact that the temporal bone had disappeared, and deconstruction was complicated by infection and inflammation caused by cholesteatoma, surgical invasion, and Gorham-Stout disease. Bony destruction was observed in other bones of the skull, including the contralateral temporal bone, mandibular bone, and zygomatic bones, which had not been affected by previous surgical procedures for cholesteatoma. Thus, an appropriate diagnosis saved the patient from having to undergo multiple ineffective surgeries for CSF leakage or cholesteatoma, thereby improving his quality of life.

## Data Availability

Not applicable.

## Abbreviations

CT: computed tomography
MRI: magnetic resonance imaging
